# Predictive value of total psoas muscle index for postoperative physical functional decline in older patients undergoing emergency abdominal surgery

**DOI:** 10.1186/s12893-023-02085-5

**Published:** 2023-06-24

**Authors:** Keishi Yamaguchi, Shokei Matsumoto, Takeru Abe, Kento Nakajima, Satomi Senoo, Masayuki Shimizu, Ichiro Takeuchi

**Affiliations:** 1 Department of Emergency Medicine, Yokohama City University Graduate School of Medicine, 4-57 Urafunecho, Minami-Ku, Yokohama-Shi, Kanagawa 232-0024 Japan; 2 Department of Trauma and Emergency Surgery, Saiseikai Yokohama-Shi Tobu Hospital, 3-6-1 Shimosueyoshi, Tsurumi-Ku, Yokohama-Shi, Kanagawa 230-0012 Japan

**Keywords:** Emergency abdominal surgery, Total psoas muscle index, Functional decline, Barthel index, Older patients

## Abstract

**Background:**

Older individuals increasingly require emergency abdominal surgeries. They are susceptible to surgical stress and loss of independence in performing daily activities. We hypothesized that the psoas muscle volume would be significantly associated with postoperative functional decline (FD) in older patients undergoing emergency abdominal surgery and aimed to evaluate the use of the psoas muscle volume on computed tomography (CT) scans.

**Methods:**

A retrospective, single-center study of patients aged ≥ 65 years who had undergone emergency abdominal surgery between January 2019 and June 2021 was performed. We assessed patients’ activities of daily living using the Barthel Index. FD was defined as a ≥ 5-point decrease between preoperative and 28-day postoperative values. The psoas muscle volume was measured by CT, which was used for diagnosis, and normalized by height to calculate total psoas muscle index (TPI). We evaluated associations between FD and TPI using receiver operating characteristics (ROC) analysis and multiple logistic regression analysis.

**Results:**

Of 238 eligible patients, 71 (29.8%) had clinical postoperative FD. Compared to the non-FD group, the FD group was significantly older and had a higher proportion of females, higher Charlson Comorbidity Index, lower body mass index, higher American Society of Anesthesiology score, lower serum albumin level, and lower TPI. ROC analyses revealed that TPI had the highest area under the curve (0.802; 95% confidence interval [CI], 0.75–0.86). A multivariable logistic regression model revealed that low TPI was an independent predictor of postoperative FD (odds ratio, 0.14; 95% CI, 0.06–0.32).

**Conclusions:**

TPI can predict postoperative FD due to emergency abdominal surgery. Identification of patients who are at high risk of FD before surgery may be useful for enhancing the regionalized system of care for emergency general surgery.

**Supplementary Information:**

The online version contains supplementary material available at 10.1186/s12893-023-02085-5.

## Background

Globally, older individuals are increasingly requiring emergency abdominal surgeries every year [1–3]. In addition to higher rates of postoperative complications, mortality, and resource utilization, these patients are more likely to lose their preoperative independence in performing activities of daily living [4, 5]. This loss of independence is a burden to the patient, their family, and the society [6]. Thus, identifying the risk factors for decreased independence may be useful in preoperative decision-making and postoperative intervention [7]. Postoperative functional decline (FD) cannot be predicted using age alone [8]; frailty, defined as a decrease of physiological reserve [9], is a greater risk factor for functional decline than age [9–11]. Several studies have reported using a preoperative frailty assessment scoring system in emergency abdominal surgery to predict postoperative FD [2, 12]. Unfortunately, acute abdominal diseases present as emergencies, and accurate medical histories and activities of daily living (ADL) are not always obtained for this assessment; therefore, a simple and effective predictive method is required.

Sarcopenia is considered a precursor to frailty [7], a risk factor for physical function decline [9]. Therefore, we hypothesized that sarcopenia could predict physical function decline earlier. Sarcopenia is useful in predicting the short-term [13, 14] and long-term [7] post-emergency laparotomy morbidity and mortality rates in older individuals. Psoas muscle volume measurement using computed tomography (CT) is reportedly a good indicator of sarcopenia [15, 16]. Abdominal CT plays a critical role in the diagnosis and preoperative management of patients with acute abdomen; the psoas muscle area can be quickly and easily measured using CT as opposed to using the scoring system. Additionally, serum albumin levels are indicators of sarcopenia in older adults [17]; FD is reportedly associated with low serum albumin levels [18].

We hypothesized that psoas muscle volume is significantly associated with FD following emergent abdominal surgeries in older patients. In this study, we measured the psoas muscle volume and various parameters at admission. This study aimed to determine whether psoas muscle volume, an indicator of sarcopenia, is more useful than other parameters in predicting postoperative FD in older patients undergoing emergency abdominal surgery.

## Methods

### Study design

We performed a retrospective analysis of our maintained emergency general surgery database (January 2019–June 2021). This noninterventional, single-center study was conducted in the Emergency and Trauma Center of Saiseikai Yokohamashi Tobu Hospital, a tertiary-care hospital in Yokohama, Japan. This observational study protocol was reviewed and approved by the institutional review board (no. 20210173). The requirement for individual written informed consent was waived by the ethics committee owing to the retrospective study design, as per the Personal Information Protection Law and National Research Ethics Guideline. The study was conducted in accordance with the principles of the Declaration of Helsinki.

This study included all consecutive patients aged ≥ 65 years who underwent emergency surgery for acute gastrointestinal abdominal pathologies between January 2019 and June 2021. Patients who died in the hospital, those who underwent gynecological surgery, those who did not undergo CT, and those who had trauma- or vascular-related abdominal diseases, elective surgical complications, and lacked the required data, such as ADL, were excluded from our study. Furthermore, patients who were bedridden (pre-morbid Barthel index score ≤ 25) were excluded because the purpose of this study was to evaluate the association between emergency abdominal surgery and FD [19].

The following data were extracted from the database: age, sex, body mass index (BMI), vital signs in the emergency room, blood test reports, discharge disposition, ADL at admission and 28 days after surgery, Charlson Comorbidity Index (CCI), and American Society of Anesthesiology (ASA) score. The blood tests included nutritional (albumin and hemoglobin), inflammatory (white blood cell count and C-reactive protein levels), tissue ischemia (lactate), and other (platelet and creatinine) markers. In addition, data on the indication for emergency surgery, surgical procedure, and operation time were extracted from the surgical database. The surgical procedures were categorized into major (defined as surgical interventions involving bowel resection, Hartmann’s procedure, or surgery for diffuse peritonitis) and intermediate-minor (defined as surgical interventions involving cholecystectomy [without diffuse peritonitis], appendectomy, adhesiolysis, stoma creation without resection, hernia repair, or diagnostic laparotomy or laparoscopy) procedures [12].

### Image analysis of the total psoas muscle area

**Fig. 1:**
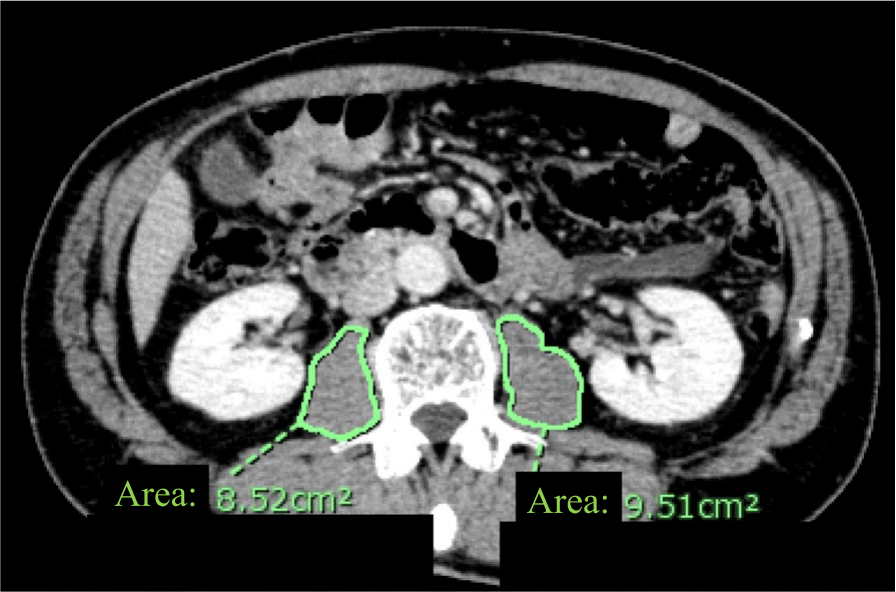
Measurement of the total psoas muscle area (TPA). The level of the third lumbar vertebra was checked and manually measured by contouring the muscle at the upper edge

Abdominal CT scans were performed using 64 multidetector CT scanners (Aquilion CT scanner; Toshiba, Tokyo, Japan) with contiguous 2-mm axial sections. Approximately 100 mL of intravenous contrast media was used for all patients unless contraindicated. The preoperative CT images were reviewed on a diagnostic Picture Archiving and Communication Systems monitor (PACS) (Shade Quest View R, Yokogawa Electric Corporation, Tokyo, Japan). To avoid observer bias, the CT scans were retrospectively reviewed and analyzed by an experienced faculty emergency radiologist (S.S.) who was blinded to patient outcomes. Psoas muscle volume was measured by obtaining the skeletal muscle cross-sectional area on CT scans as previously described [7, 14, 15]. The third lumbar (L3) vertebral level was identified, and the total psoas muscle area (TPA) was measured at the superior margin of the L3 vertebra by manually outlining the muscle (Fig. 1). The areas of the right and left psoas muscles were measured in cm^2^ and averaged. TPA was normalized by height to calculate the total psoas muscle index (TPI = TPA/height^2^; cm^2^/m^2^), which was used for statistical analysis [7]. Additionally, psoas muscle density, an indication of muscle quality, was measured using the Hounsfield unit average calculation (HU), cautiously excluding bones or foreign objects within the measurement area [13].

### Evaluation of ADL and FD

The Barthel index, reportedly the best ADL assessment scale, measures the degree of independence of patients in mobility and personal care, and was used for ADL evaluation in our study [20, 21]. This index was scored by the rehabilitation team at the time of admission and 28 days after surgery. Evaluation on admission was performed by the rehabilitation team preoperatively based on information from the individual or family member before surgery. However, in some cases, when the team was not available before surgery, especially at night, patients were evaluated at the day after surgery. If patients were discharged or transferred within these 28 days, the assessment was conducted via interview of a family member or facility staff.

The Barthel index is a reliable and accurate measure of autonomy in performing ADL and is sensitive to functional capacity changes [22, 23]. The items on the Barthel index are related to self-care (eating, grooming, bathing, dressing, defecation/urination, and using the toilet) and mobility (walking, moving, and climbing stairs). The scale ranges from 0 (for a completely dependent, bedridden state) to 100 (for a completely independent state); since the minimum change is a 5-point increase or decrease, Barthel index score with a decrease of ≥ 5 points is considered FD [22].

In accordance with our hospital’s clinical practice program, an individualized post-surgical rehabilitation program, designed by a multidisciplinary team, was started for all patients, unless there was a specific contraindication. The rehabilitation team comprised a rehabilitation physician, physiotherapists, and nurses. The program included 30–60 min of daily activities conducted 5 days per week.

### Statistical analysis

The study variables were compared between the functional (FD group) and non-functional (non-FD group) decline groups. Continuous variables are expressed as medians with interquartile ranges and categorical variables as whole numbers. A univariable analysis of the patient variables was performed using the chi-square test, Fisher’s exact test, Student’s t-test, Mann–Whitney test, or paired t-test, as appropriate. Model discrimination was assessed by calculating the area under the receiver operating characteristic (ROC) curve (AUC). Moreover, ROC analyses defined the best cut-off points for these continuous variables as the maximum sum of sensitivity and specificity (Youden index) [24]. The potential predictive risk factors, including age, sex, BMI, serum albumin levels, ASA score, CCI, and surgical interventions, were screened using a univariable logistic model (*p* < 0.05). Subsequently, multiple logistic regression analysis was performed to identify the risk factors for FD in older patients after abdominal surgery; TPI was screened in addition to the previously screened risk factors. We evaluated multicollinearity using the variance inflation factor and added quadratic terms to check for polynomial relationships. Statistical significance was set at *p* < 0.05. Odds ratios (ORs) were calculated with the corresponding 95% confidence intervals (CIs). All statistical analyses were performed using R 4.1.1 (R Core Team (2021). R: A language and environment for statistical computing. R Foundation for Statistical Computing, Vienna, Austria. URL https://www.R-project.org/).

## Results

During the 30-month study period, 741 patients required emergency abdominal surgery, 327 of whom were aged over 65 years; of these patients, 238 met the criteria and were eligible for the study (see Additional file 1: Appendix 1, Supplemental Digital Content).

**Fig. 2:**
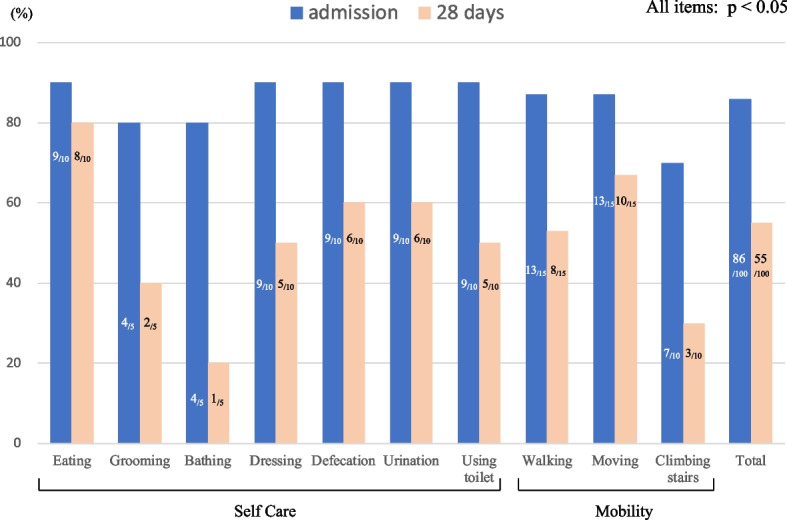
Barthel index breakdown in FD patients. The numbers in the graphs indicate the score for each item. Significant decline is seen in all categories

The FD and non-FD groups included 71(29.8%) and 167 (70.2%) patients, respectively. The baseline characteristics of both groups are presented in Table 1. Overall, the median length of hospital stay was 12 (interquartile range, 8–23) days, and the most common disease was cholecystitis (19.3%), followed by strangulated intestinal obstruction (15.1%), bowel perforation (13.9%), and appendicitis (12.2%). The median length of hospital stay was significantly longer in the FD group (23 vs 11 days, *p* < 0.001) than in the non-FD group, and patients in the FD group were significantly less likely to be discharged home (46.5% vs 94.0%, *p* < 0.001). Figure 2 shows the Barthel index breakdown at the time of admission and 28 days after surgery in FD patients. Because each item on the index has a different maximum score, ranging from 5 to 15, Fig. 2 is presented as a percentage. All items were significantly decreased at 28 days than at admission. Among them, bathing was decreased most in self-care (75% drop) and climbing stairs was decreased most in mobility (57% drop).

**Table 1:** Patient characteristics stratified by their functional decline status

Characteristic	Functional decline *n* = 71	Non–functional decline *n* = 167	*P* value
Patient variable			
Age, median (IQR)	83 (78–88)	75 (71–81)	< 0.001
Female sex, n (%)	46 (64.8)	65 (38.9)	< 0.001
BMI: median (IQR)			
kg/m^2^	20.8 (18.8–23.5)	22.2 (19.5–24.5)	0.028
Vital signs, median (IQR)			
Respiratory rate			
/min	19 (17–24)	18 (16–20)	0.105
Heart rate			
/min	88 (76–107)	86 (73–97)	0.086
Systolic blood pressure			
mmHg	132 (104–151)	132 (116–155)	0.252
Body temperature			
℃	36.7 (36.4–37.2)	36.6 (36.3–37.0)	0.508
CCI ≥ 4, n, (%)	62 (87.3)	92 (55.1)	< 0.001
Diagnosis, n (%)			0.003
Cholecystitis	10 (14.1)	36 (21.6)	
Strangulated intestinal obstruction	12 (16.9)	24 (14.4)	
Bowel perforation	9 (12.7)	24 (14.4)	
Appendicitis	0 (0)	29 (17.4)	
Complicated hernia	10 (14.1)	16 (9.6)	
Malignant obstruction	5 (7.0)	13 (7.8)	
Acute mesenteric ischemia	10 (14.1)	5 (3.0)	
Adhesive intestinal obstruction	5 (7.0)	7 (4.2)	
Gastric/Duodenal perforation	5 (7.0)	4 (2.4)	
Others	5 (7.0)	9 (5.4)	
Outcome			
Hospital LOS, median (IQR)			
days	23 (14–36)	11 (2–16)	< 0.001
Discharge disposition, n (%)			< 0.001
Home	33 (46.5)	157 (94.0)	
Others	38 (53.5)	10 (6.0)	

*IQR* Interquartile range, *BMI* Body mass index, *CCI* Charlson Comorbidity Index, *LOS* Length of stay

### Relationship between functional decline and predictors

#### Clinical characteristics and vital signs

The median (interquartile range) age of the patients was 78 (72–84) years, with 22.3% of them aged ≥ 85 years and 53.4% of them being men. A comparison of the clinical characteristics between the FD and non-FD groups is shown in Table 1. The FD group was significantly older (median age, 83 vs 75 years, *p* < 0.001), had a higher CCI (4 vs 3, *p* < 0.001), higher proportion of women (64.8% vs 38.9%, *p* < 0.001), and a lower BMI (20.8 vs 22.2, *p* = 0.028) than the non-FD group. However, vital signs were similar between the two groups.

#### Blood tests and surgical procedures

The comparison of blood test results and surgical procedures between the two groups is summarized in Table 2. The FD group had significantly lower albumin levels (3.3 g/dL vs 3.9 g/dL, *p* < 0.001) than the non-FD group. However, there was no significant difference in other blood test results between the two groups. No patient in the FD group presented with appendicitis, and the ASA scores were significantly higher in this group (3 vs 2, *p* < 0.001). More major surgeries were performed in the FD group than in the non-FD group (59.2% vs 37.1%, *p* = 0.003).

**Table 2:** Blood tests, details of surgery, and total psoas muscle index

Characteristic	Functional decline *n* = 71	Non–functional decline *n* = 167	*P* value
Blood tests, median (IQR)			
pH	7.40 (7.36–7.45)	7.41 (7.37–7.43)	0.667
Lactate			
mg/dL	19 (12–38)	16 (11–24)	0.079
Albumin			
g/dL	3.3 (2.8–3.7)	3.9 (3.2–4.3)	< 0.001
White blood cell			
× 1000/μL	9.1(6.7–13)	10.7 (7.2–15)	0.143
Platelet			
× 10000/μL	22.3 (17.3–27.6)	21.2 (17.3–27.4)	0.934
Hemoglobin			
g/dL	12.8 (10.5–15.1)	13.5 (12.2–15.3)	0.108
Creatine			
mg/dL	1.1 (0.7–1.9)	0.9 (0.7–1.2)	0.051
C-reactive protein			
mg/dL	4.6 (0.9–13)	3.1 (0.2–13)	0.171
Details of surgery			
ASA score, n, (%)			< 0.001
I	1 (1.4)	12 (7.2)	
II	22 (31.0)	109 (65.3)	
III	42 (59.2)	42 (25.1)	
IV	6 (8.5)	4 (2.4)	
Operation			
Time, median (IQR)			
min	110 (69.0–146.5)	111 (76.5–154.0)	0.599
Surgical intervention, n (%)			0.003
Major	42 (59.2)	62 (37.1)	
Intermediate-Minor	29 (40.8)	105 (62.9)	
Total psoas muscle index (IQR)			
Total psoas muscle index			
cm^2^/m^2^	1.84 (1.48–2.13)	2.46 (1.99–2.96)	< 0.001
Density of psoas muscle			
HU	40.3 (30.8–46.2)	38.4 (31.4–45.7)	0.618

*IQR* Interquartile range, *ASA* American Society of Anesthesiology, *HU* Hounsfield units average calculation

#### Total psoas muscle index and predictors of functional decline

**Fig. 3:**
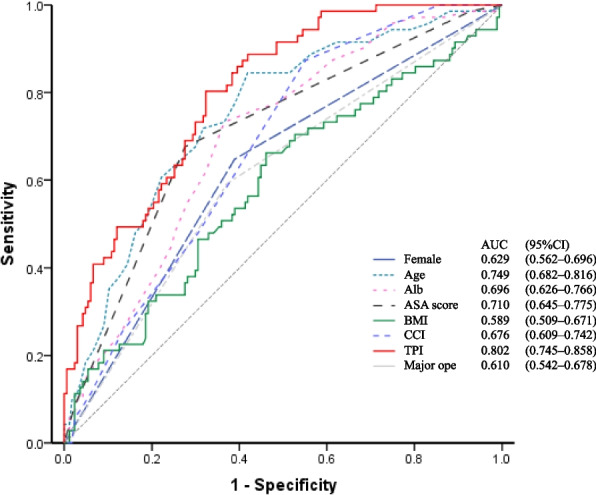
Area under the receiver operating characteristic curve for the items. TPI, total psoas muscle index; ASA, American Society of Anesthesiology; Alb, Albumin; CCI, Charlson Comorbidity Index

Further, Table 2 summarizes the TPI. The results were significantly lower in the FD group (1.84 vs 2.46, *p* < 0.001). No difference was found in psoas density between the two groups. Figure 3 shows the AUC of the items that were significant in the univariable analysis. The AUC was highest for TPI in the FD prognosis (AUC, 0.802; 95% CI, 0.75–0.86). The TPI was evaluated as an FD prognostic test with an optimal cut-off point, sensitivity, specificity, and negative and positive predictive values of 67.7%, 80.3%, 89.0%, and 51.4%, respectively (see Additional file 2: Appendix 2, Supplemental Digital Content). In the multivariable logistic regression model, TPI, ASA score, and age were significantly associated with FD (OR, 0.14, 95% CI, 0.06–0.32; OR, 3.83, 95% CI, 1.89–7.76; and OR, 1.14, 95% CI, 1.07–1.21, respectively) (Table 3).

**Table 3:** Univariable and multivariable logistic regression analyses

Factor	Univariable analysis			Multivariable analysis		
	**OR**	**95% CI**	***p***	**OR**	**95% CI**	***p***
Age	1.13	(1.09–1.18)	< 0.001	1.14	(1.07–1.21)	< 0.001
Albumin	0.39	(0.26–0.59)	< 0.001	0.56	(0.31–1.02)	0.058
ASA score	3.75	(2.29–6.15)	< 0.001	3.83	(1.89–7.76)	< 0.001
BMI	0.93	(0.87–1.01)	0.077		–	
CCI	1.45	(1.20–1.77)	< 0.001	0.88	(0.64–1.19)	0.399
Female	2.89	(1.62–5.15)	< 0.001	1.62	(0.70–3.77)	0.263
Major operation	2.45	(1.39–4.33)	0.002	1.39	(0.64–3.00)	0.405
TPI	0.11	(0.06–0.22)	< 0.001	0.14	(0.06–0.32)	< 0.001

*ASA* American Society of Anesthesiology, *BMI* Body mass index, *CCI* Charlson Comorbidity Index, *TPI* Total psoas muscle index, *OR* Odds ratio, *CI* Confidence interval

Thus, the TPI can most likely predict FD. However, the physical features of men and women are very different. Therefore, an interaction analysis of the effect of TPI and sex was performed. No interaction was observed between the two sex-based groups (*p* = 0.61; see Additional file 3: Appendix 3, Supplemental Digital Content).

## Discussion

The global proportion of older individuals is steadily increasing. In developed countries, the average life expectancy has been increasing and is now well over 80 years [25]; this is associated with an increased number of emergency surgeries in this age group [1–3]. Unfortunately, emergency surgery may promote FD requiring nursing care [26]. As a result, FD in older individuals is becoming common and creating a burden on these individuals, their families, and the society [6]. Thus, detecting and preventing FD at an early stage in cases of emergency surgery are necessary. In this study, we hypothesized that sarcopenia could predict physical function decline and identified the predictive factors of postoperative FD based on the preoperative clinical findings, including TPI, age, and ASA scores, using a multivariate regression analysis. The strongest predictive factor was the TPI with the ROC analyses (AUC, 0.802).

In the initial management for patients with acute abdomen, a CT scan is often performed for diagnosis. Therefore, the TPI can be calculated without additional testing. In emergencies, the image is zoomed simultaneously with the diagnosis, and the psoas muscles can be manually outlined to the left and right (using only the visual thresholding process of PACS), and the area can be calculated promptly.

The most crucial outcomes for older patients requiring emergency surgery are reduction in FD and preoperative physical function maintenance aside from morbidity and mortality [4, 27]. Therefore, FD prediction is critical; however, only a few studies have been conducted regarding this. Frailty has been identified as a strong predictor of decreased independence 1 year postoperatively [9]. A few frailty identification tools exist; however, they are complex and may not always be feasible in a busy emergency room setting [9]. The Flemish version of the Triage Risk Screening Tool (fTRST), a simple frailty tool, can effectively predict the FD in a population of older patients undergoing emergency abdominal surgery [12]. In this study, fTRST ≥ 2 showed the highest prognostic value with AUC of 0.72. We did not score fTRST in our study because of information shortage; obtaining this information is difficult because in Japan, several senior citizens live alone. Conversely, CT and blood test findings are objective, easily interpreted, and are now considered essential for acute abdominal disease. Additionally, low serum albumin levels have been identified as an FD risk factor in older patients hospitalized for infections [18]. In the present study, the FD group had significantly lower albumin levels; however, the difference was not statistically significant in the multivariate analysis. Although serum albumin can provide a benchmark for sarcopenia, blood tests are less likely to provide a prognostic value for FD [17].

Furthermore, in this study, we clarified FD characteristics using the Barthel index. Associations between emergency abdominal surgery and FD characteristics have not been previously examined. This study found that “bathing” (in self-care) and “climbing stairs” (in mobility) were the most affected. The iliopsoas muscles, the main hip joint flexors, are crucial for movements such as “bathing” and “climbing stairs”; therefore, in this study, a low TPI may be easily reflected in FD, which is defined as a Barthel index score change. Identifying patients with a predicted FD preoperatively using the TPI and considering early home support for FD after discharge, especially for “bathing” and “climbing stairs,” may contribute to improving the quality of life (QOL).

In recent years, sarcopenia is one of the most important preoperative considerations. Sarcopenia reportedly increases postoperative adverse events and mortality in elective surgery [28–30]. Additionally, in older patients undergoing emergency abdominal surgery, sarcopenia is associated with increased postoperative complications, prolonged hospital stays, and increased mortality [7, 14, 15, 31]. The TPI is frequently used as an indicator of sarcopenia [7, 32–35]. TPI measurement is simple and objective and can be used in an emergency setting where efficiency and practicality are crucial. Since there is no accepted standard value for TPI, previous studies have generally defined sarcopenia when the TPI is in the lowest quartile of sex difference [7, 32–34, 36]. While previous studies have defined a low TPI differently for men and women, the interaction analysis in this study found no effect of sex on the FD. This may be due to the lower muscle-volume difference between men and women in Asian populations than that in Caucasian populations [37, 38] and the study findings of a lower sex-based difference when adjusted for height [39].

To the best of our knowledge, this is the first study to examine the association between psoas muscle volume and postoperative FD in emergency surgery. Patients with sarcopenia defined by a low TPI reportedly demonstrate high perioperative risk and poor short-term and long-term survival outcomes after emergency abdominal surgery [7, 13, 35]. The present study showed that a low TPI was significantly associated with postoperative FD. In elective surgery, better preoperative nutrition and physical activity improve prognosis [40]. However, in emergency surgery, preoperative interventions are difficult to implement because of the narrow time window before surgery. For patients with a low TPI, more attention should be paid to less-invasive medical treatments, early postoperative nutrition, rehabilitation, complication prevention, and intensive care. Preoperative identification of low TPI could provide important predictive information for not only medical management strategies but also family awareness [41]. Our findings regarding an association between low TPI and postoperative FD after emergency abdominal surgery may be novel and partly consistent with previous findings. Just as physical function enhancement improves the prognosis of elective surgery [40], FD prevention may play a critical role in improving the prognosis of emergency surgery.

This study had several limitations. First, this was a retrospective, single-center study. Therefore, the generalizability of our findings is limited. Although this study was conducted among Asian participants, the association between low TPI and FD in other racial groups is unknown. Second, since this study examined whether postoperative physical FD could be predicted using preoperative information, selection bias may have existed owing to the lack of homogeneity in postoperative complications. Future studies should consider the association between postoperative complications and FD. Third, the manual TPI measurement in this study may have led to errors. Furthermore, this study did not assess the factors related to the performing surgeons, including their backgrounds and experiences. Finally, family support during hospitalization and after discharge was not considered, which may have affected the postoperative functional status. Despite these limitations, we believe that our findings will provide a firm foundation for future studies.

In conclusion, the TPI is a potential predictive factor for postoperative FD following emergency abdominal surgery as it can be measured quickly without any additional testing. Identification of patients who are at high risk of FD before surgery may be useful for enhancing the regionalized system of care for emergency general surgery.

## Supplementary Information

**Additional file 1.** Patient flow in this study.

**Additional file 2.** Accuracy of each factor as a predictive test for functional decline.

**Additional file 3.** Interaction analysis between the effect of total psoas muscle index and sex.

## Data Availability

The datasets generated and/or analysed during the current study are not publicly available due to unethical restriction by IRB, but are available from the corresponding author on reasonable request.

## Abbreviations

ADL: Activities of daily living
ASA: American Society of Anesthesiology
AUC: Area under the receiver operating characteristic curve
BMI: Body mass index
CCI: Charlson Comorbidity Index
CIs: Confidence intervals
CT: Computed tomography
FD: Functional decline
fTRST: Flemish version of the Triage Risk Screening Tool
HU: Hounsfield Units
ORs: Odds ratios
PACS: Picture Archiving and Communication Systems monitor
QOL: Quality of life
ROC: Receiver operating characteristic
ROC: Receiver operating characteristics
TPA: Total psoas muscle area
TPI: Total psoas muscle index

## References

[1] St-Louis E, Sudarshan M, Al-Habboubi M, El-Husseini Hassan M, Deckelbaum DL, Razek TS (2016). The outcomes of the elderly in acute care general surgery. Eur J Trauma Emerg Surg.

[2] Desserud KF, Veen T, Søreide K (2016). Emergency general surgery in the geriatric patient. Br J Surg.

[3] Ukkonen M, Jämsen E, Zeitlin R, Pauniaho SL (2019). Emergency department visits in older patients: a population-based survey. BMC Emerg Med.

[4] Fried TR, Bradley EH, Towle VR, Allore H (2002). Understanding the treatment preferences of seriously ill patients. N Engl J Med.

[5] Joseph B, Zangbar B, Pandit V, Kulvatunyou N, Haider A, O’Keeffe T (2014). Mortality after trauma laparotomy in geriatric patients. J Surg Res.

[6] McIsaac DI, Moloo H, Bryson GL, van Walraven C (2017). The association of frailty with outcomes and resource use after emergency general surgery: a population-based cohort study. Anesth Analg.

[7] Rangel EL, Rios-Diaz AJ, Uyeda JW, Castillo-Angeles M, Cooper Z, Olufajo OA (2017). Sarcopenia increases risk of long-term mortality in elderly patients undergoing emergency abdominal surgery. J Trauma Acute Care Surg.

[8] Merani S, Payne J, Padwal RS, Hudson D, Widder SL, Khadaroo RG (2014). Predictors of in-hospital mortality and complications in very elderly patients undergoing emergency surgery. World J Emerg Surg.

[9] Tan HL, Chia STX, Nadkarni NV, Ang SY, Seow DCC, Wong TH (2019). Frailty and functional decline after emergency abdominal surgery in the elderly: a prospective cohort study. World J Emerg Surg.

[10] Farhat JS, Velanovich V, Falvo AJ, Horst HM, Swartz A, Patton JH (2012). Are the frail destined to fail? Frailty index as predictor of surgical morbidity and mortality in the elderly. J Trauma Acute Care Surg.

[11] Joseph B, Pandit V, Zangbar B, Kulvatunyou N, Hashmi A, Green DJ (2014). Superiority of frailty over age in predicting outcomes among geriatric trauma patients: a prospective analysis. JAMA Surg.

[12] Zattoni D, Montroni I, Saur NM, Garutti A, Bacchi Reggiani ML, Ghignone F (2021). Prediction of functional loss in emergency surgery is possible with a simple frailty screening tool. World J Emerg Surg.

[13] Dirks RC, Edwards BL, Tong E, Schaheen B, Turrentine FE, Shada A (2017). Sarcopenia in emergency abdominal surgery. J Surg Res.

[14] Brandt E, Tengberg LT, Bay-Nielsen M (2019). Sarcopenia predicts 90-day mortality in elderly patients undergoing emergency abdominal surgery. Abdom Radiol (NY).

[15] Hamidi M, Ho C, Zeeshan M, O’Keeffe T, Hamza A, Kulvatunyou N (2019). Can sarcopenia quantified by computed tomography scan predict adverse outcomes in emergency general surgery?. J Surg Res.

[16] Janssen I, Heymsfield SB, Ross R (2002). Low relative skeletal muscle mass (sarcopenia) in older persons is associated with functional impairment and physical disability. J Am Geriatr Soc.

[17] Baumgartner RN, Koehler KM, Romero L, Garry PJ (1996). Serum albumin is associated with skeletal muscle in elderly men and women. Am J Clin Nutr.

[18] Nakano H, Hashimoto H, Mochizuki M, Naraba H, Takahashi Y, Sonoo T (2020). Hypoalbuminemia on admission as an independent risk factor for acute functional decline after infection. Nutrients.

[19] Rozzini R, Sabatini T, Cassinadri A, Boffelli S, Ferri M, Barbisoni P (2005). Relationship between functional loss before hospital admission and mortality in elderly persons with medical illness. J Gerontol A Biol Sci Med Sci.

[20] Tasheva P, Vollenweider P, Kraege V, Roulet G, Lamy O, Marques-Vidal P (2020). Association between physical activity levels in the hospital setting and hospital-acquired functional decline in elderly patients. JAMA Netw Open.

[21] Shah S, Vanclay F, Cooper B (1989). Improving the sensitivity of the Barthel index for stroke rehabilitation. J Clin Epidemiol.

[22] Fimognari FL, Pierantozzi A, De Alfieri W, Salani B, Zuccaro SM, Arone A (2017). The severity of acute illness and functional trajectories in hospitalized older medical patients. J Gerontol A Biol Sci Med Sci.

[23] Palleschi L, De Alfieri W, Salani B, Fimognari FL, Marsilii A, Pierantozzi A (2011). Functional recovery of elderly patients hospitalized in geriatric and general medicine units. The PROgetto DImissioni in GEriatria study. J Am Geriatr Soc.

[24] Akobeng AK (2007). Understanding diagnostic tests 3: Receiver operating characteristic curves. Acta Paediatr.

[25] Kontis V, Bennett JE, Mathers CD, Li G, Foreman K, Ezzati M (2017). Future life expectancy in 35 industrialised countries: projections with a Bayesian model ensemble. Lancet.

[26] Torrance AD, Powell SL, Griffiths EA (2015). Emergency surgery in the elderly: challenges and solutions. Open Access Emerg Med.

[27] Fried TR, Tinetti ME, Iannone L, O’Leary JR, Towle V, Van Ness PH (2011). Health outcome prioritization as a tool for decision making among older persons with multiple chronic conditions. Arch Intern Med.

[28] Wang PY, Xu LD, Chen XK, Xu L, Yu YK, Zhang RX (2020). Sarcopenia and short-term outcomes after esophagectomy: a meta-analysis. Ann Surg Oncol.

[29] Shen Y, Hao Q, Zhou J, Dong B (2017). The impact of frailty and sarcopenia on postoperative outcomes in older patients undergoing gastrectomy surgery: a systematic review and meta-analysis. BMC Geriatr.

[30] Ratnayake CB, Loveday BP, Shrikhande SV, Windsor JA, Pandanaboyana S (2018). Impact of preoperative sarcopenia on postoperative outcomes following pancreatic resection: a systematic review and meta-analysis. Pancreatology.

[31] Hajibandeh S, Hajibandeh S, Jarvis R, Bhogal T, Dalmia S (2019). Meta-analysis of the effect of sarcopenia in predicting postoperative mortality in emergency and elective abdominal surgery. Surgeon.

[32] Englesbe MJ, Patel SP, He K, Lynch RJ, Schaubel DE, Harbaugh C (2010). Sarcopenia and mortality after liver transplantation. J Am Coll Surg.

[33] Peng P, Hyder O, Firoozmand A, Kneuertz P, Schulick RD, Huang D (2012). Impact of sarcopenia on outcomes following resection of pancreatic adenocarcinoma. J Gastrointest Surg.

[34] Joglekar S, Asghar A, Mott SL, Johnson BE, Button AM, Clark E (2015). Sarcopenia is an independent predictor of complications following pancreatectomy for adenocarcinoma. J Surg Oncol.

[35] Yang TR, Luo K, Deng X, Xu L, Wang RR, Ji P (2022). Effect of sarcopenia in predicting postoperative mortality in emergency laparotomy: a systematic review and meta-analysis. World J Emerg Surg.

[36] Amini N, Spolverato G, Gupta R, Margonis GA, Kim Y, Wagner D (2015). Impact total psoas volume on short- and long-term outcomes in patients undergoing curative resection for pancreatic adenocarcinoma: a new tool to assess sarcopenia. J Gastrointest Surg.

[37] Baumgartner RN, Koehler KM, Gallagher D, Romero L, Heymsfield SB, Ross RR (1998). Epidemiology of sarcopenia among the elderly in New Mexico. Am J Epidemiol.

[38] Sanada K, Miyachi M, Tanimoto M, Yamamoto K, Murakami H, Okumura S (2010). A cross-sectional study of sarcopenia in Japanese men and women: reference values and association with cardiovascular risk factors. Eur J Appl Physiol.

[39] Melton LJ, Khosla S, Crowson CS, O’Connor MK, O’Fallon WM, Riggs BL (2000). Epidemiology of sarcopenia. J Am Geriatr Soc.

[40] Whittle J, Wischmeyer PE, Grocott MPW, Miller TE (2018). Surgical prehabilitation: nutrition and exercise. Anesthesiol Clin.

[41] Susano MJ, Grasfield RH, Friese M, Rosner B, Crosby G, Bader AM (2020). Brief preoperative screening for frailty and cognitive impairment predicts delirium after spine surgery. Anesthesiology.

