# Supplementary figures and images for: Predictive value of total psoas muscle index for postoperative physical functional decline in older patients undergoing emergency abdominal surgery
Source: BMC Surg. 2023 Jun 24;23:171. doi: 10.1186/s12893-023-02085-5 (PMC10290795; doi:10.1186/s12893-023-02085-5)

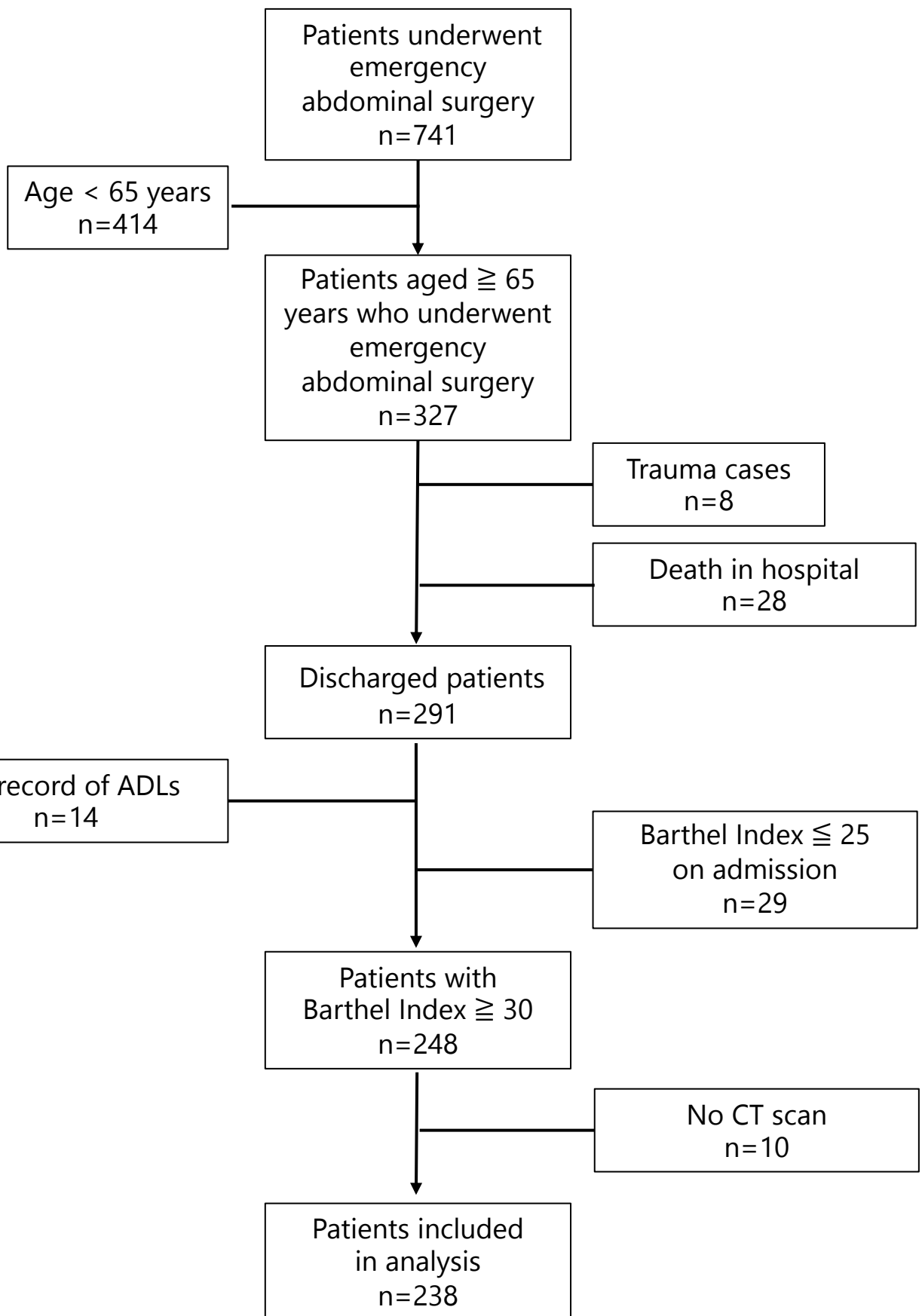

Appendix 1: Patient flow in this study

Supplement: Supplementary file 1 — Additional file 1. Patient flow in this study. [file 12893_2023_2085_MOESM1_ESM.pdf]

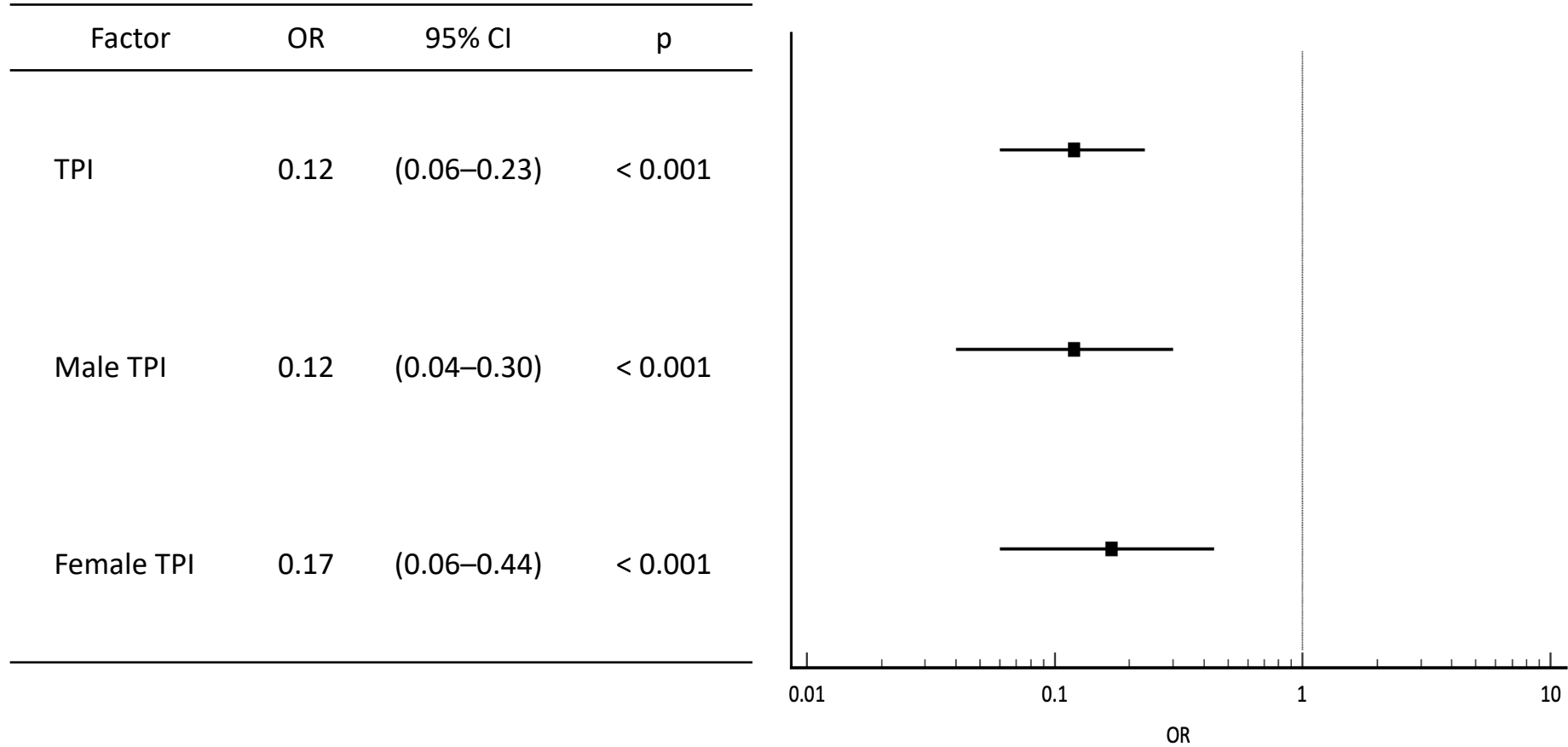

Appendix 3: Interaction analysis between the effect of total psoas muscle index and sex

Supplement: Supplementary file 3 — Additional file 3. Interaction analysis between the effect of total psoas muscle index and sex. [file 12893_2023_2085_MOESM3_ESM.pdf]
