# Supplementary material for: Predictive value of total psoas muscle index for postoperative physical functional decline in older patients undergoing emergency abdominal surgery
Source: BMC Surg. 2023 Jun 24;23:171. doi: 10.1186/s12893-023-02085-5 (PMC10290795; doi:10.1186/s12893-023-02085-5)
Supplement: Supplementary file 2 — Additional file 2. Accuracy of each factor as a predictive test for functional decline. [file 12893_2023_2085_MOESM2_ESM.docx]

Appendix 2. Accuracy of each factor as a predictive test for functional decline

| **Factor** | | **Cut-off point** | **AUC** | **95% CI** | **Sensitivity** | **Specificity** | **NPV** | **PPV** |
| --- | --- | --- | --- | --- | --- | --- | --- | --- |
| ASA score | | > 2 | 0.710 | (0.645–0.775) | 72.5 | 67.6 | 84.0 | 51.1 |
| Age | | > 76 | 0.749 | (0.682–0.816) | 58.1 | 84.5 | 89.8 | 46.2 |
| Albumin | (g/dL) | < 3.7 | 0.696 | (0.626–0.766) | 63.9 | 73.2 | 84.8 | 46.0 |
| BMI | (kg/m^2^) | < 22.1 | 0.590 | (0.509–0.671) | 53.3 | 66.2 | 78.8 | 37.6 |
| CCI | | > 3 | 0.676 | (0.609–0.742) | 44.9 | 87.3 | 89.3 | 40.3 |
| TPI | (cm^2^/m^2^) | < 2.16 | 0.802 | (0.745–0.858) | 67.7 | 80.3 | 89.0 | 51.4 |

ASA, American Society of Anesthesiology; BMI, body mass index; CCI, Charlson Comorbidity Index; TPI, total psoas muscle index; AUC, area under the receiver operating characteristic curve; CI, confidence interval; NPV, negative predictive value; PPV, positive predict value
